# Biodiversity of non-*Saccharomyces* yeasts associated with spontaneous fermentation of Cabernet Sauvignon wines from Shangri-La wine region, China

**DOI:** 10.1038/s41598-021-83216-x

**Published:** 2021-03-04

**Authors:** Yue Zhao, Qingyang Sun, Shusheng Zhu, Fei Du, Ruzhi Mao, Lijing Liu, Bin Tian, Yifan Zhu

**Affiliations:** 1 College of Plant Protection, Yunnan Agricultural University, Kunming, 650201 China; 2 College of Food Science and Technology, Yunnan Agricultural University, Kunming, 650201 China; 3 University Engineering Research Center for Grape and Wine of Yunan Province, Yunnan Agricultural University, Kunming, 650201 China; 4 Faculty of Agriculture and Life Sciences, Lincoln University, Lincoln, 7647 New Zealand

**Keywords:** Microbiology, Molecular biology

## Abstract

Shangri-La is a wine region that has the highest altitude vineyards in China. This is the first study investigated the biodiversity of non-*Saccharomyces* yeasts associated with spontaneous fermentation of Cabernet Sauvignon wines produced from two sub-regions (Lancang River and Jinsha River) of Shangri-La. The culturable yeasts were preliminarily classified based on their colonial morphology on the Wallerstein Laboratory nutrient agar plates. Yeast species were identified by the sequencing of the 26S rRNA D1/D2 region and the 5.8S rRNA ITS region. Twenty-five non-*Saccharomyces* yeast species belonging to sixteen genera were isolated and identified in Shangri-La wine region. *Candida*, *Hanseniaspora*, *Pichia,* and *Starmerella* were found in both sub-regions, but the Lancang River showed more diverse yeast species than the Jinsha River. Shangri-La not only exhibited high diversity of non-*Saccharomyces* yeasts, and furthermore, seven species of non-*Saccharomyces* yeasts were exclusively found in this region, including *B. bruxellensis, D. hansenii, M. guilliermondii*, *S. vini*, *S. diversa*, *T. delbrueckii* and *W. anomalus*, which might play an important role in distinctive regional wine characteristics. This study provide a relatively comprehensive analysis of indigenous non-*Saccharomyces* yeasts associated with Cabernet Sauvignon from Shangri-La, and has significance for exploring ‘microbial *terroir*’ of wine regions in China.

## Introduction

Wine fermentation is a complex biochemical process conducted by many different microorganisms, and yeasts play a major role in this process^1^. Although wine could be produced spontaneously by wild yeasts on the surface of grapes^2^, inoculation of commercial *Sacchromyces cerevisiae* yeast is the most common operation in current wine production in order to avoid several potential problems (*e.g.* sluggish fermentation) and to achieve final products with uniform quality^3^. According to some opinions, the resulting wine, however, is more like ‘industrial’ products and loses its ‘natural’ property such as diversity and distinctive characteristics^2^.

To produce wine with distinctive characteristics, researchers and enologists tried to inoculate non-*Saccharomyces* yeasts during alcoholic fermentation^4–7^. In these studies, non-*Saccharomyces* yeasts were either co-inoculated or sequentially inoculated with *S. cerevisiae* yeast, and the resultant wines generally exhibited more varietal characteristics and distinctive sensory attributes. In addition, non-*Saccharomyces* yeasts have also been used in the production of other beverages, such as beer^8^ and spirit^9,10^. Although a few non-*Saccharomyces* yeasts are commercially available, there is an increasing interest in exploring non-*Saccharomyces* yeasts worldwide and their potential usage in wine production.

Some research have demonstrated that indigenous yeasts are an important part of the ‘*terroir*’ in different wine producing regions around the world^11–14^. The density and diversity of those indigenous yeasts on grape are closely related to numerous factors, such as the variety and maturity of grape, geographical location and climatic conditions of vineyard, and the practices of viticulture^11^. China has a fast-growing wine industry which includes numerous wine regions. These regions are located mostly between northern latitudes 35° and 40°, except for the Shangri-La wine region which is located between northern latitudes 28° and 30°^15^. Shangri-La is a wine region rich in biodiversity attributed to the complex topography. It has a typical dry and hot valley climate with timely dry season from October/November until June of the following year. It has adequate sunlight with high intensity of ultraviolet and large diurnal temperature variation^16^. This distinctive climate could produce wines with high quality and regional characteristics, which has attracted great interests of wine producers and researchers. In recent years, some research have studied the density and diversity of indigenous yeast population in several wine regions of China, such as Helan-mountains, Qilian-mountains, Huai-Zhuo basin and Bohai bay et al.^17–21^. Yeast species identified in those wine regions are generally within genera *Candida*, *Hanseniaspora*, *Issatchenkia*, *Metschnikowia* and *Pichia*. However, limited study has been carried out focusing on the indigenous yeast in Shangri-La wine region, particularly non-*Saccharomyces* yeasts.

Therefore, the aim of this study was to investigate the biodiversity of non-*Saccharomyces* yeasts associated with spontaneous fermentation of Cabernet Sauvignon wines from Shangri-La wine region and obtain regional characteristic non-*Saccharomyces* yeast species. This study could improve our understanding on the ‘microbial *terroir*’ of Shangri-La wine region, and provide potential yeast resources for distinctive wine production.

## Results

### Oenological parameters in grape juice and spontaneously fermented wine

The oenological parameters in Cabernet Sauvignon grape juice and spontaneously fermented wines from Shangri-La were shown in Table 1. Grapes from each vineyard exhibited various ripeness with the reducing sugar concentration ranging from 186.74 to 265.95 g/L, total acidity ranging from 3.42 to 8.55 g/L, and the pH ranging from 3.26 to 3.97. After spontaneous fermentation, most of wines were fermented to dryness with residual sugar concentration less than 5.00 g/L, except for wines made from L-XD (10.91 g/L) and J-BZL (14.14 g/L). Due to the variations in ripeness of grapes and the residual sugar in wines, the alcohol content of resultant wines ranged between 10.79 and 15.46%. There was an increase in the total acidity during the spontaneous fermentation. The concentration of volatile acidity varied significantly among the wines ranging from 0.60 to 1.92 g/L. The pH of final sample wines varied from 3.38 to 3.84.

**Table 1:** Oenological parameters in Cabernet Sauvignon grape juice and spontaneously fermented wine. Concentration values (mean ± SD, n = 3) of the same parameter followed by different letters are significantly different (*p* < 0.05) employing Duncan multiple range tests.

Sample ID	Grape juice	Spontaneously fermented wine						
	Reducing sugar (g/L)	Total acidity (tartaric acid g/L)	pH (20 °C)	Residual sugar (g/L)	Alcohol (% V/V)	Total acidity (tartaric acid g/L)	Volatile acidity (acetic acid g/L)	pH (20 °C)
L-NT	186.74 ± 0.97e	8.55 ± 0.05a	3.26	1.77 ± 0.23f.	10.79 ± 0.13f.	9.04 ± 0.06a	0.79 ± 0.03de	3.38
L-XD	265.95 ± 1.32a	3.85 ± 0.05e	3.74	10.91 ± 0.03b	15.38 ± 0.02a	6.21 ± 0.06e	1.22 ± 0.02c	3.67
L-LTJ	265.16 ± 0.86a	3.57 ± 0.02f.	3.85	3.67 ± 0.01d	15.46 ± 0.05a	6.02 ± 0.02f.	0.82 ± 0.02d	3.66
L-SN	236.06 ± 0.75d	4.27 ± 0.02d	3.44	4.46 ± 0.02c	13.70 ± 0.09c	8.15 ± 0.02b	0.72 ± 0.04e	3.44
L-AD	239.56 ± 0.94c	4.60 ± 0.01b	3.49	2.22 ± 0.02e	12.79 ± 0.09e	7.26 ± 0.01c	0.60 ± 0.01f.	3.57
J-BZL	252.64 ± 2.22b	3.42 ± 0.05g	3.97	14.14 ± 0.16a	14.15 ± 0.05b	7.25 ± 0.06c	1.92 ± 0.09a	3.76
J-DR	239.12 ± 2.03c	4.34 ± 0.02c	3.73	2.24 ± 0.56e	13.38 ± 0.03d	6.56 ± 0.05d	1.47 ± 0.06b	3.84

### Yeast isolation and identification

**Figure 1:**
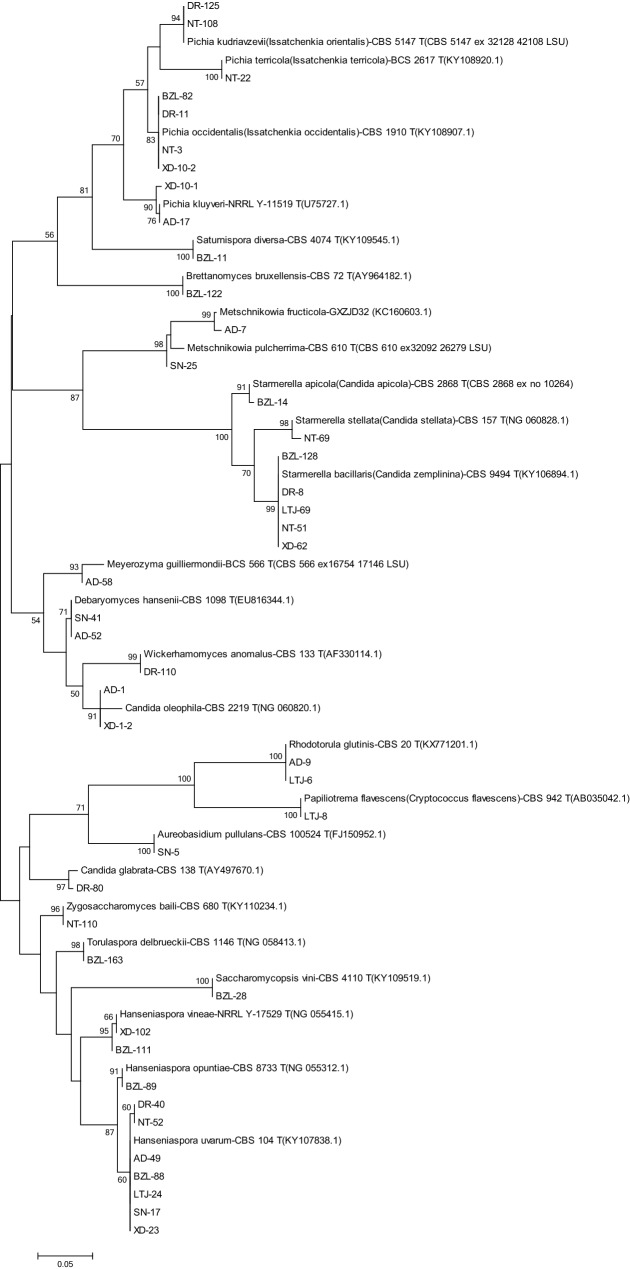
Phylogenetic tree of representative non-*Saccharomyces* yeast isolates obtained from Shangri-La wine region based on the sequence analysis of the 26S rRNA D1/D2 region using the maximum-likelihood method. The scale bar shows 0.05, Bootstrap support values were estimated based on 1000 replicates and are shown above the branches (> 50%).

**Figure 2:**
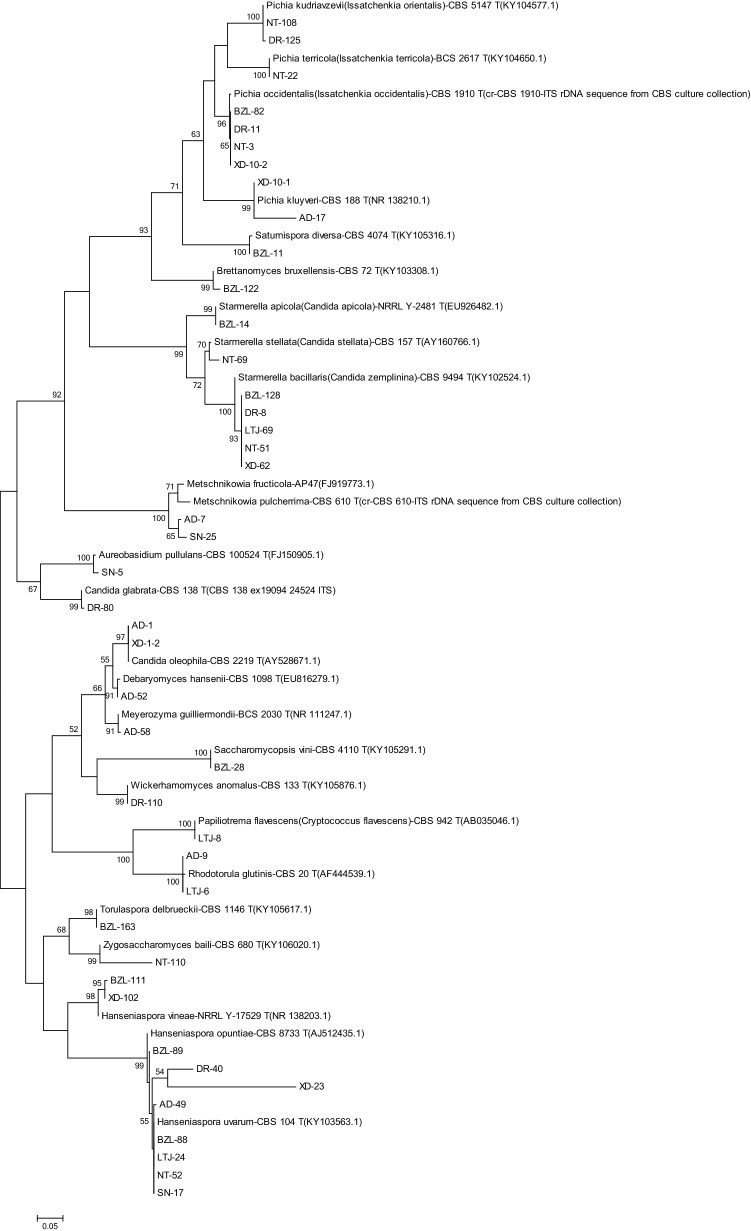
Phylogenetic tree of representative non-*Saccharomyces* yeast isolates obtained from Shangri-La wine region based on the sequence analysis of the 5.8S rRNA ITS region using the maximum-likelihood method. The scale bar shows 0.05, Bootstrap support values were estimated based on 1000 replicates and are shown above the branches (> 50%).

The detailed information of 26S rRNA D1/D2 region and 5.8S rRNA ITS region of representative isolates were listed in Table S1. Most yeast isolates were identified at high similarity (> 99.00%) of D1/D2 and ITS sequences with the corresponding type strains. The reliable identification results were also confirmed by phylogenetic analysis (Figs. 1, 2). Although a high similarity was shown in BLAST results of 26S rRNA D1/D2 region, certain differences were observed in 5.8S rRNA ITS region among several isolates and related type strains, including L-SN-25, L-AD-7, L-AD-17, L-XD-23 and L-NT-69 (Table S1). As no type strain is available for *Metschnikowia fructicola*, the identification of L-AD-7 was based on BLAST analysis of 26S rRNA D1/D2 region of GXZJD32 (KC160603.1) and 5.8S rRNA ITS region of AP47 (FJ919773.1) (Table S1).

### Biodiversity of non-Saccharomyces yeasts in Shangri-La region

A total of 2168 yeast isolates were collected during spontaneous fermentation of Cabernet Sauvignon wines from seven samples within the Lancang and Jinsha River Basins of Shangri-La wine region. As shown in Table 2, twenty-five different non-*Saccharomyces* yeast species belonging to sixteen genera were identified by colonial morphology and molecular methods. The identified yeast species include *Aureobasidium pullulans*, *Brettanomyces bruxellensis*, *Candida glabrata*, *Candida oleophila*, *Debaryomyces hansenii*, *Hanseniaspora opuntiae*, *Hanseniaspora uvarum*, *Hanseniaspora vineae*, *Metschnikowia fructicola*, *Metschnikowia pulcherrima*, *Meyerozyma guilliermondii*, *Papiliotrema flavescens* (*Cryptococcus flavescens*), *Pichia kluyveri*, *Pichia kudriavzevii* (*Issatchenkia orientalis*), *Pichia occidentalis* (*Issatchenkia occidentalis*), *Pichia terricola* (*Issatchenkia terricola*), *Rhodotorula glutinis*, *Saccharomycopsis vini*, *Saturnispora diversa*, *Starmerella apicola* (*Candida apicola*), *Starmerella bacillaris* (*Candida zemplinina*), *Starmerella stellata* (*Candida stellata*), *Torulaspora delbrueckii*, *Wickerhamomyces anomalus* and *Zygosaccharomyces bailii*. There were more non-*Saccharomyces* yeast species isolated from the Lancang River than the Jinsha River, and the common genera found in both sub-regions were *Candida*, *Hanseniaspora*, *Pichia* and *Starmerella*. The sample of J-BZL showed more non-*Saccharomyces* yeast diversity than other vineyards in Shangri-La region (Table 2). According to the data from other wine regions of China, Shangri-La region showed higher overall diversity of non-*Saccharomyces* yeasts. Furthermore, seven species of non-*Saccharomyces* yeasts are exclusively found in Shangri-La, including *B. bruxellensis, D. hansenii, M. guilliermondii*, *S. vini*, *S. diversa*, *T. delbrueckii* and *W. anomalus*.

**Table 2:** Frequency of non-*Saccharomyces* yeast isolates from Cabernet Sauvignon spontaneous fermentation of Shangri-La wine region. ‘−’ means yeast species was not detected in present work.

Yeast species	L-NT	L-XD	L-LTJ	L-SN	L-AD	J-BZL	J-DR	Representative isolates
*Aureobasidium pullulans*	–	–	–	2.7	–	–	–	SN-5
*Brettanomyces bruxellensis*	–	–	–	–	–	4.7	–	BZL-122
*Candida glabrata*	–	–	–	–	–	–	7.2	DR-80
*Candida oleophila*	–	1.1	–	–	1.2	–	–	XD-1-2, AD-1
*Debaryomyces -hansenii*	–	–	–	6.6	2.4	–	–	SN-41, AD-52
*Hanseniaspora opuntiae*	–	–	–	–	–	2.9	–	BZL-89
*Hanseniaspora uvarum*	19.7	27.2	40.2	38.5	28.4	20.2	16.8	NT-52, XD-23, LTJ-24, SN-17, AD-49, BZL-88, DR-40
*Hanseniaspora vineae*	–	9.8	–	–	–	2.5	–	XD-102, BZL-111
*Metschnikowia fructicola*	–	–	–	–	4.3	–	–	AD-7
*Metschnikowia pulcherrima*	–	–	–	2.7		–	–	SN-25
*Meyerozyma guilliermondii*	–	–	–	–	5.0	–	–	AD-58
*Papiliotrema flavescens* (*Cryptococcus flavescens*)	–	–	4.9	–	–	–	–	LTJ-8
*Pichia kluyveri*	–	2.3	–	–	2.6	–	–	XD-10-1, AD-17
*Pichia kudriavzevii* (*Issatchenkia orientalis*)	6.6	–	–	–	–	–	2.3	NT-108, DR-125
*Pichia occidentalis* (*Issatchenkia occidentalis*)	3.3	2.3	–	–	–	5.2	2.9	NT-3, XD-10-2, BZL-82, DR-11
*Pichia terricola* (*Issatchenkia terricola*)	3.0	–	–	–	–	–	–	NT-22
*Rhodotorula glutinis*	–	–	5.4	–	3.8	–	–	LTJ-6, AD-9
*Saccharomycopsis vini*	–	–	–	–	–	2.5	–	BZL-28
*Saturnispora diversa*	–	–	–	–	–	1.1	–	BZL-11
*Starmerella apicola* (*Candida apicola*)	–	–	–	–	–	1.8	–	BZL-14
*Starmerella bacillaris* (*Candida zemplinina*)	15.8	6.4	7.4	–	–	6.7	15.1	NT-51, XD-62, LTJ-69, BZL-128, DR-8
*Starmerella stellate* (*Candida stellata*)	2.0	–	–	–	–	–	–	NT-69
*Torulaspora delbrueckii*	–	–	–	–	–	3.8	–	BZL-163
*Wickerhamomyces anomalus*	–	–	–	–	–	–	5.2	DR-110
*Zygosaccharomyces bailii*	1.6	–	–	–	–	–	–	NT-110
*Saccharomyces cerevisiae*	48.0	50.9	42.2	49.5	52.5	48.5	50.4	–

## Discussion

Compared to most wine regions of China^19^, the Cabernet Sauvignon harvested from Shangri-La exhibited greater maturity degree, which was related to the ideal viticulture environment of this region16. In most spontaneously fermented wines, the concentration of volatile acidity were much higher than acceptable level (0.7 g/L)^22^. Indeed, most non-*Saccharomyces* yeasts^23–26^ and some wild *S. cerevisiae* yeasts^19,25^ may produce excess volatile acidity during spontaneous fermentation. While a marked increase of total acidity in final wines suggested that some yeast species being found in present study could be used for improving the wine contained insufficient acidity^27^.

Within the sixteen non-*Saccharomyces* yeast genera identified in Shangri-La region, *Hanseniaspora* and *Starmerella* were the most frequent isolates. The predominant species of these two genera were *H. uvarum* and *S. bacillaris* (*C. zemplinina*), which is in agreement with the findings of previous research^11,12,18,28^. These two species were commonly found in grape and wine, thus more attention has been paid in vinification research and application^29–32^ In addition, four members of *Hanseniaspora* and *Starmerella* also existed in Shangri-La region, namely *H. opuntiae*, *H. vineae*, *S. apicola* (*C. apicola*) and *S. stellata* (*C. stellata*).

The *Pichia* is another abundant genus associated with spontaneous fermentation of Cabernet Sauvignon in Shangri-La region. Among the four *Pichia* species isolated, *P. occidentalis* was the most abundant species. Interestingly, *P. occidentalis* had also been found in Roussanne that grown in Beijing^17^. While for other wine regions of China, the widely distributed species of this genus was *Pichia fermentans*^18,21^*.* Several studies indicated that some *Pichia* species, such as *P. kluyveri*, *P. kudriavzevii*(*I. orientalis*), *P. terricola*(*I. terricola*) and *Pichia fermentans* have the potential to enhance wine aroma by releasing glycosidically bound aroma precursors from grape must^4,33^ or producing volatile compounds during fermentation^5,34,35^. But there is little information about *P. occidentalis* (*I. occidentalis*) in wine production, which might be worth further study.

Two *Metschnikowia* species, including *M. fructicola* and *M. pulcherrima*, have been isolated in the samples of L-AD and L-SN, respectively. *M. pulcherrima* was the common species that had been found on Cabernet Sauvignon among most wine regions of China^18–21^. Although *M. fructicola* had previously only been isolated on Roussanne in Beijing^17^, the present study indicated that it may also associated with Cabernet Sauvignon that grown in China. According to studies conducted by Floriana Boscaino^36^, Elena González-Royo^6^ and C. Varela^37^, the usage of *M*. *fructicola* or *M*. *pulcherrima* during fermentation could improve the aromatic profile of resulatnt wine.

In this study, *P. flavescens* (*C. flavescens*) was only accounted for a small proportion in the sample of L-LTJ. Although a previous study reported that *P. flavescens* (*C. flavescens*) was found as a common yeast species isolated from Cabernet Sauvignon, Merlot and Chardonnay in Shacheng, Changli, Wuwei and Penglai of China^18^, this species seems unlikely associate with grape due to the low frequency observed in this study, which was in agreement with the view of Brysch-Herzberg^12^.

*A. pullulans* (so called black-yeast) and *R. glutinis*, which have the capacity of pigment metabolism, were found in present work. *A. pullulans* is one of the most well-adapted saprophytes on grape berries^20^, however the distribution of *A. pullulans* was not very widespread in Shangri-La as well as other regions of China^18–21^. It was only detected in sample of L-SN and exhibited a low isolation frequency. It has been reported that various enzymes (*e.g.* pectinase, cellulases and *β*-glucosidase) produced by *A. pullulan* could be beneficial to the flavor of wine^38^. Additionally, due to antifungal and antibacterial activity, this species might be used to against some biological diseases of vine^39^. Although *R. glutinis* were accidentally isolated in two samples of Shangri-La, *Rhodotorula* sp. are generally considered as typical phyllospheric yeasts^40^ and thus rarely associated with yeast community of grape berries in China^18–21^. Although some *Rhodotorula* sp. can produce *β*-glucosidase and *α*-l-arabinofuranosidase to release bound aroma precurcors^41,42^, they are rarely used in wine production.

*Zygosacchromyces bailii* was found in the sample of L-NT. It is one of the most dangerous wine spoilage species which are known to produce off-flavors and form cloudiness even under high alcohol condition^43,44^. Although *Z. bailii* exhibited low isolation frequency in Shangri-La, some precautions should be taken in wine production.

There were seven non-*Saccharomyces* yeast species which showed regional characteristics were exclusively found in Shangri-La wine region, including *B. bruxellensis, D. hansenii, M. guilliermondii*, *T. delbrueckii, W. anomalus*, *S. vini* and *S. diversa*. To the best of our knowledge, this is the first time that these non-*Saccharomyces* yeast species have been isolated from wine regions of China^17–21^. *T. delbrueckii* is one of the commercialized non-*Saccharomyces* yeast for wine production. Several positive influences of *T. delbrueckii*, such as enhancing the fruity aroma style, increasing glycerol concentration, reducing volatile acidity and improving foam properties, have been confirmed in the production of grape^6,45^ and other fruit wines^46,47^. In this study, J-BZL-163 (*T. delbrueckii*) was isolated from the plateau area with unique environmental conditions. Therefore, further research will be required to evaluate the differences of fermentation characteristics between J-BZL-163 and commercial *T. delbrueckii* strain. Although *B. bruxellensis* is generally considered as spoilage microorganisms, it could also contribute distinctive attributes in spirits^9^. *W. anomalus* and *D. hansenii* have also been tested in wine-related research and shown positive effects on wine aroma quality^48,49^*.* Although *M. guilliermondii*, *S. vini* and *S. diversa* have been found in wine^50^, beer^51^ and some fruits^52,53^, little study has been conducted for fermentation characteristics of these yeasts in wine or other alcoholic beverages.

Compared with other wine regions of China that have been reported^17–21^, Shangri-La wine region exhibited greater overall diversity of non-*Saccharomyces* yeast species. Within Shangri-La region, J-BZL showed more non-*Saccharomyces* yeast diversity than other vineyards, which is most probably related to its distinctive grapegrowing environment. Compared to other six vineyards in Shangri-La region, the vineyard of J-BZL is very close to the bank of Jinsha River, which provides a high humidity environment ideal for epiphytic microorganism growth on grapes^54^. As for L-LTJ and L-SN which located at the foot of Meili Snow Mountain, a rapid decline in temperature during mature period (October) could be one of the possibilities that reduce the diversity of non-*Saccharomyces* yeasts present on their grapes. According to França’s research, soil samples that came from low temperature area exhibited less density of yeast population. Furthermore, low temperature may also make a few yeast become the dominated culturable species and reduce the chance that other species will be isolated^55^. These results confirmed that the geographic locations and climate conditions have great impact on yeast communities^13^ and provided more evidence that indigenous yeasts should be considered as an important part of ‘*terroir*’^14^.

## Methods

### Grape samples and spontaneous fermentation

**Figure 3:**
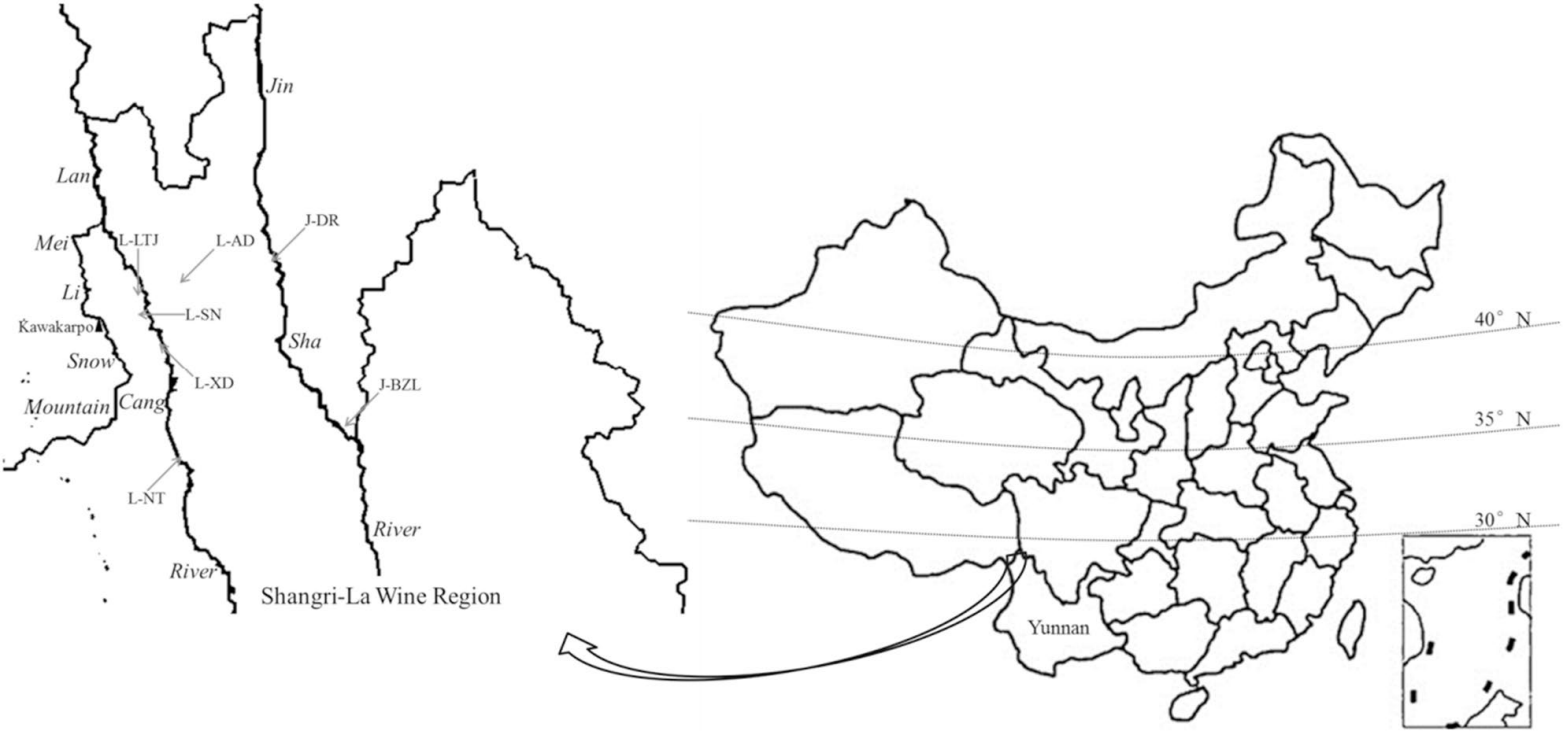
The location of sampling vineyards from Shangri-La wine region.

Cabernet Sauvignon (*Vitis vinifera* L.) grape samples were collected from seven vineyards (Shangri-La Winery Co., Ltd. Yunnan) located at different altitudes within two sub-regions (Lancang River and Jinsha River) of Shangri-La wine region in Yunan province. Grape samples were harvested by hand with sterile gloves in September and October in 2017. After harvest, grape samples (10 kg) from each vineyard were stored in sterile bags at 4 °C and transported to the laboratory in Kunming within 24 h. The geographical information and the location of sampling vineyards was summarized and shown in Table 3 and Fig. 3, respectively.

**Table 3:** The geographical information of sampling vineyards from Shangri-La wine region. *In sample ID, L- and J-indicates sample collected from Lancang River and Jinsha River in Shangri-La wine region, respectively.

Sub-region	Sample ID*	Altitude (m)	Latitude	Longitude	Sample date
Lancang River	L-NT	1942	28°08′10″	98°53′18″	2017.09.08
	L-XD	2081	28°26′55″	98°49′35″	2017.09.08
	L-LTJ	2121	28°33′20″	98°47′13″	2017.10.14
	L-SN	2266	28°29′38″	98°48′7″	2017.10.14
	L-AD	2602	28°33′45″	98°52′12″	2017.10.14
Jinsha River	J-BZL	2009	28°13′10″	99°19′20″	2017.09.08
	J-DR	2238	28°35′35″	99°10′37″	2017.09.08

Grape samples of each vineyard were destemmed and crushed by hand with sterile gloves, and the must was fermented in two sterilized 5 L glass bottles at 28 °C for 12–18 days. During the fermentation, cap management was carried out once a day until the end of fermentation. Oenological parameters in Cabernet Sauvignon grape juice (reducing sugar concentration, total acidity, and pH) and spontaneously fermented wines (residual sugar concentration, total acidity, volatile acidity, pH and alcohol content) were measured according to the National Standard of the People's Republic of China: GB/T 15,038–2006, “Analytical Methods of Wine and Fruit Wine”. The concentration of reducing/residual sugar was determined by 3,5-dinitrosalicylic acid method. The content of alcohol was determined by density method. The concentration of total acidity was determined by potentiometric titration using standard sodium hydroxide. The separation of volatile acidity from wine was carried out by steam distillation. The concentration of volatile acidity was titrated by standard sodium hydroxide. The value of pH was determined by pH meter.

### Isolation of non-Saccharomyces yeasts

For the isolation of non-*Saccharomyces* yeasts, fermenting samples (10 ml) were collected every 3 days from each fermentation bottle. Due to the variation in the fermentation duration, there were 4 fermenting samples collected from L-LTJ, L-SN; 5 fermenting samples collected from L-NT, L-XD and J-DR; 6 fermenting samples collected from L-AD and J-BZL. The fermenting samples were diluted into 1:10^3^ to 1:10^5^ ratios with sterile physiological solution (0.85%, NaCl), plated on the Wallerstein Laboratory (WL) nutrient agar (Qingdao Hope Bio-Technology Co., Ltd.) and incubated at 28 °C for 5 days until colonies showed morphological differences. About 10 to 15 colonies showing different colonial morphology were selected from each WL nutrient agar plate, and then re-streaked on the YEPD agar plate (yeast extract 10 g/L, tryptone 20 g/L, glucose 20 g/L, agar 20 g/L, and chloramphenicol 100 mg/L) for purification. There were 2168 pure yeast cultures in total isolated and stored at 4 °C for further analysis. Glycerol (15% v/v) was added into yeast culture for long-term storage at -80 °C.

### Non-Saccharomyces yeasts identification

Yeast isolates were preliminarily classified according to the morphology (color, shape, consistency, and size) of their colonies on the WL nutrient agar plate, and a total of 204 isolates were chosen for molecular identification.

Genomic DNA was extracted from three colonies of each isolates by using Rapid Yeast Genomic DNA Isolation Kit (Shanghai Sangon Biotech Co., Ltd.) following the manufacturer’s instructions. The genomic DNA extracted was identified by analysis of the sequence similarity of the 26S rRNA D1/D2 region and 5.8S rRNA ITS region. The primers for amplification of 26S rRNA D1/D2 region were NL1 (5′-GCATATCAATAAGCGGAGGAAAAG-3′) and NL4 (5′-GGTCCGTGTTTCAAGACGG-3′)^56^, and for amplification of 5.8S rRNA ITS region were ITS1 (5′-TCCGTAGGTGAACCTGCGG-3′) and ITS4 (5′-TCCTCCGCTTATTGATATGC-3′)^57^. All the sample DNAs were amplified in PCRs at a 40 μL reaction volume, containing 2 µL of extracted DNA, 2 µL of each primer (100 μmol/L), 20 µL of Master Mix (ABM) and 14 µL of sterile double distilled H_2_O. PCR amplification was carried out in an Applied Biosystems, Veriti 96-well Thermocycler, and the PCR thermal cycling conditions were as follows: 5 min at 95 °C; 36 cycles of 1 min at 94 °C, 1 min at 55 °C, 1 min at 72 °C; and finally 10 min at 72 °C.

The PCR products were purified and sequenced by Biomed gene technology Co., Ltd (Beijing). The sequencing results were submitted and compared with those of corresponding type strain (listed in Supplementary Table S1) in the Blast service of the National Center for Biotechnology Information (NCBI http://www.ncbi.nlm.nih.gov/blast).

Phylogenetic analysis was performed to verify the identification of representative isolates. The phylogenetic tree was developed using the Maximum Likelihood methods and the Kimura 2-parameter model by MEGA 7.0: Molecular Evolutionary Genetics Analysis version 7.0 for bigger datasets (Kumar, Stecher, and Tamura 2015). Bootstrap values were calculated from 1000 iterations.

### Statistical analysis

All the analyses were performed in triplicate. One-way analysis of variance (ANOVA) was used to analyze the difference of grape and wine samples performed through SPSS 19.0 (IBM Corp., Armonk, New York, U.S.A.) employing Duncan multiple range tests at a significance level of *p* < 0.05. The results were expressed as the mean value ± the standard deviation.

## Supplementary Information

Supplementary Information.

## References

[1] Fugelsang KC, Edwards CG (2007). Grape and wine microorganisms. Wine microbiology.

[2] Jolly NP, Varela C, Pretorius IS (2014). Not your ordinary yeast: Non-*Saccharomyces* yeasts in wine production uncovered. FEMS Yeast Res..

[3] Padilla, B., Gil J. V. & Manzanares P. Past and future of non-*Saccharomyces* yeasts: From spoilage microorganisms to biotechnological tools for improving wine aroma complexity. *Front. Microbiol*. **7,** Article 411 (2016). 10.3389/fmicb.2016.00411 PMC4814449 27065975

[4] Anfang N, Brajkovich M, Goddard MR (2009). Co-fermentation with *Pichia kluyveri* increases varietal thiol concentrations in Sauvignon Blanc. Aust. J. Grape Wine Res..

[5] Benito S (2015). Effect on quality and composition of Riesling wines fermented by sequential inoculation with non-*Saccharomyces* and *Saccharomyces cerevisiae*. Eur. Food Res. Technol..

[6] González-Royo E (2015). Oenological consequences of sequential inoculation with non-*Saccharomyces* yeasts (*Torulaspora delbrueckii* or *Metschnikowia pulcherrima*) and *Saccharomyces cerevisiae* in base wine for sparkling wine production. Eur. Food Res. Technol..

[7] Ivit NN, Loira I, Morata A, Benito S, Palomero F, Suárez-Lepe JA (2017). Making natural sparkling wines with non-*Saccharomyces yeasts*. Eur. Food Res. Technol..

[8] Toh DWK, Chua JY, Liu SQ (2018). Impact of simultaneous fermentation with *Saccharomyces cerevisiae* and *Torulaspora delbrueckii* on volatile and non-volatile constituents in beer. LWT Food Sci. Technol..

[9] Parente DC, Vidal EE, Leite FCB, Pita WBP, Morais MA (2015). Production of sensory compounds by means of the yeast *Dekkera bruxellensis* in different nitrogen sources with the prospect of producing cachaça. Yeast.

[10] Amaya-Delgado L, Herrera-López EJ, Arrizon J, Arellano-Plaza M, Gschaedler A (2013). Performance evaluation of *Pichia kluyveri*, *Kluyveromyces marxianus* and *Saccharomyces cerevisiae* in industrial tequila fermentation. World J. Microbiol. Biotechnol..

[11] Barata A, Malfeito-Ferreira M, Loureiro V (2012). The microbial ecology of wine grape berries. Int. J. Food Microbiol..

[12] Brysch-Herzberg M, Seidel M (2015). Yeast diversity on grapes in two German wine growing regions. Int. J. Food Microbiol..

[13] Gayevskiy V, Goddard MR (2012). Geographic delineations of yeast communities and populations associated with vines and wines in New Zealand. ISME J..

[14] Bokulich NA, Thorngate JH, Richardson PM, Mills DA (2014). Mmicrobial biogeography of wine grapes is conditioned by cultivar, vintage, and climate. Proc. Natl. Acad. Sci. U.S.A..

[15] Xing RR, He F, Xiao HL, Duan CQ, Pan QH (2016). Accumulation pattern of flavonoids in Cabernet Sauvignon grapes grown in a low-latitude and high-altitude region. S. Afr. J. Enol. Vitic..

[16] Yue TX (2015). Aroma characterization of Cabernet Sauvignon wine from the plateau of Yunnan (China) with different altitudes using SPME-GC/MS. Int. J. Food Prop..

[17] Sun HH, Ma HQ, Hao ML, Pretorius IS, Chen SW (2008). Identification of yeast population dynamics of spontaneous fermentation in Beijing wine region China. Ann. Microbiol..

[18] Li SS (2010). Yeast species associated with wine grapes in China. Int. J. Food Microbiol..

[19] Li EH, Liu AG, Xue B, Liu YL (2011). Yeast species associated with spontaneous wine fermentation of Cabernet Sauvignon from Ningxia, China. World J. Microbiol. Biotechnol..

[20] Sun Y, Guo JJ, Liu FB, Liu YL (2014). Identification of indigenous yeast flora isolated from the five winegrape varieties harvested in Xiangning, China. Antonie van Leeuwenhoek.

[21] Liu PT, Lu L, Duan CQ, Yan GL (2016). The contribution of indigenous non-*Saccharomyces* wine yeast to improved aromatic quality of Cabernet Sauvignon wines by spontaneous fermentation. LWT Food Sci. Technol..

[22] Lambrechts MG, Pretorius IS (2000). Yeast and its importance to wine aroma. S. Afr. J. Enol. Vitic...

[23] Sadoudi M (2012). Yeast-yeast interactions revealed by aromatic profile analysis of Sauvignon Blanc wine fermented by single or co-culture of non-*Saccharomyces* and *Saccharomyces* yeasts. Food Microbiol..

[24] Jolly NP, Augustyn OPH, Pretorius IS (2003). The effect of non-*Saccharomyces* yeasts on fermentation and wine quality. S. Afr. J. Enol. Vitic...

[25] Domizio P, Romani C, Lencioni L, Comitini F, Ciani M (2011). Outlining a future for non-*Saccharomyces* yeasts: Selection of putative spoilage wine strains to be used in association with *Saccharomyces cerevisiae* for grape juice fermentation. Int. J. Food Microbiol..

[26] Mendoza LM, Vega-Lopez GA, Ullivarri MF, Raya RR (2019). Population and oenological characteristics of non-*Saccharomyces* yeasts associated with grapes of Northwestern Argentina. Arch. Microbiol..

[27] Gobbi M (2013). *Lachancea thermotolerans* and *Saccharomyces cerevisiae* in simultaneous and sequential co-fermentation: A strategy to enhance acidity and improve the overall quality of wine. Food Microbiol..

[28] Drumonde-Neves J, Franco-Duarte R, Lima T, Schuller D, Pais C (2017). Association between Grape Yeast Communities and the Vineyard Ecosystems. PLoS ONE.

[29] Englezos V (2016). *Starmerella bacillaris* and *Saccharomyces cerevisiae* mixed fermentations to reduce ethanol content in wine. Appl. Microbiol. Biotechnol..

[30] Englezos V (2016). Aroma profile and composition of Barbera wines obtained by mixed fermentations of *Starmerella bacillaris* (synonym *Candida zemplinina*) and *Saccharomyces cerevisiae*. LWT Food Sci. Technol..

[31] Moreira N, Mendes F, Guedes de Pinho T, Hogg T, Vasconcelos I (2008). Heavy sulphur compounds, higher alcohols and esters production profile of *Hanseniaspora uvarum* and *Hanseniaspora guilliermondii* grown as pure and mixed cultures in grape must. Int. J. Food Microbiol..

[32] Hong YA, Park HD (2013). Role of non-*Saccharomyces* yeasts in Korean wines produced from Campbell early grapes: Potential use of *Hanseniaspora uvarum* as a starter culture. Food Microbiol..

[33] González-Pombo P, Fariña L, Carrau F, Batista-Viera F, Brena BM (2011). A novel extracellular *β*-glucosidase from *Issatchenkia terricola*: Isolation, immobilization and application for aroma enhancement of white muscat wine. Process Biochem..

[34] Mónaco SM, Barda NB, Rubio NC, Caballero AC (2014). Selection and characterization of a Patagonian *Pichia kudriavzevii* for wine deacidification. J. Appl. Microbiol..

[35] Kong CL, Li AH, Su J, Wang XC, Chen CQ, Tao YS (2019). Flavor modification of dry red wine from Chinese spine grape by mixed fermentation with *Pichia fermentans* and *S. cerevisiae*. LWT Food Sci. Technol..

[36] Boscaino F, Ionata E, Cara FL, Guerriero S, Marcolongo L, Sorrentino A (2019). Impact of *Saccharomyces cerevisiae* and *Metschnikowia fructicola* autochthonous mixed starter on Aglianico wine volatile compounds. J. Food Sci. Technol..

[37] Varela C, Sengler F, Solomon M, Curtin C (2016). Volatile flavour profile of reduced alcohol wines fermented with the non-conventional yeast species *Metschnikowia pulcherrima* and *Saccharomyces uvarum*. Food Chem..

[38] Prasongsuk S, Lotrakul P, Ali I, Bankeeree W, Punnapayak H (2017). The current status of *Aureobasidium pullulans* in biotechnology. Folia Microbiol..

[39] Dimakopoulou M (2008). Phyllosphere grapevine yeast *Aureobasidium pullulans* reduces *Aspergillus carbonarius* (sour rot) incidence in wine-producing vineyards in Greece. Biol. Control.

[40] Rosa C, Péter G (2006). Phylloplane Yeasts. The Yeasts Handbook-Biodiversity and Ecophysiology of Yeasts.

[41] Hu K, Zhu XL, Mu H, Ma Y, Ullah N, Tao YS (2016). A novel extracellular glycosidase activity from *Rhodotorula mucilaginosa*: Its application potential in wine aroma enhancement. Lett. Appl. Microbiol..

[42] Martínez C, Gertosio C, Labbe A, Pérez R, Ganga MA (2006). Production of *Rhodotorula glutinis*: A yeast that secretes *α*-L-arabinofuranosidase. Electron. J. Biotechnol..

[43] Fleet GH (2003). Yeast interactions and wine flavour. Int. J. Food Microbiol..

[44] Loureiro V, Malfeito-Ferreira M (2003). Spoilage yeasts in the wine industry. Int. J. Food Microbiol..

[45] Renaultab P, Coulonb J, Revelac G, Barbeac JC, Belya M (2015). Increase of fruity aroma during mixed *T. delbrueckii*/*S. cerevisiae* wine fermentation is linked to specific esters enhancement. Int. J. Food Microbiol..

[46] Sun SY, Gong HS, Zhao YP, Liu WL, Jin CW (2016). Sequential culture with *Torulaspora delbrueckii* and S*accharomyces cerevisiae* and management of fermentation temperature to improve cherry wine quality. J. Sci. Food Agric..

[47] Lu YY, Voon MKW, Chua JY, Huang DJ, Lee PR, Liu SQ (2017). The effects of co- and sequential inoculation of Torulaspora delbrueckii and Pichia kluyveri on chemical compositions of durian wine. Appl. Microbiol. Biotechnol..

[48] Riccio P (1999). Extraction and immobilization in one step of two *β*-glucosidases released from a yeast strain of *Debaryomyces hansenii*. Enzyme Microbial Technol..

[49] Cañas PMI, García-Romero E, Manso JMH, Fernández-González M (2014). Influence of sequential inoculation of *Wickerhamomyces anomalus* and *Saccharomyces cerevisiae* in the quality of red wines. Eur. Food Res. Technol..

[50] Varela C (2016). The impact of non-*Saccharomyces* yeasts in the production of alcoholic beverages. Appl. Microbiol. Biotechnol..

[51] Spitaels F (2014). The microbial diversity of traditional spontaneously fermented Lambic beer. PLoS ONE.

[52] Gamero A, Quintilla R, Groenewald M, Alkema W, Boekhout T, Hazelwood L (2016). High-throughput screening of a large collection of non-conventional yeasts reveals their potential for aroma formation in food fermentation. Food Microbiol..

[53] Lorenzini M, Simonato B, Zapparoli G (2018). Yeast species diversity in apple juice for cider production evidenced by culture-based method. Folia Microbiol..

[54] Rousseau S, Donèche B (2001). Effects of water activity (a_w_) on the growth of some epiphytic micro-organisms isolated from grape berry. Vitis..

[55] França L, Sannino C, Turchetti B, Buzzini P, Margesin R (2016). Seasonal and altitudinal changes of culturable bacterial and yeast diversity in Alpine forest soils. Extremophiles Life Under Extreme Cond..

[56] Kurtzman CP, Robnett CJ (1998). Identification and phylogeny of ascomycetous yeasts from analysis of nuclear large subunit (26s) ribosomal DNA partial sequences. Antonie Van Leeuwenhoek.

[57] Souza Libera TD, Silva Filho EA, Morais JOF, Simões DA, Morais MA (2005). Contaminant yeast detection in industrial ethanol fermentation must by rRNA-PCR. Lett. Appl. Microbiol..

