# Supplementary material for: Biodiversity of non-Saccharomyces yeasts associated with spontaneous fermentation of Cabernet Sauvignon wines from Shangri-La wine region, China
Source: Sci Rep. 2021 Mar 4;11:5150. doi: 10.1038/s41598-021-83216-x (PMC7933366; doi:10.1038/s41598-021-83216-x)
Supplement: Supplementary file 1 — Supplementary Information. [file 41598_2021_83216_MOESM1_ESM.docx]

**Biodiversity of non-*Saccharomyces* yeasts associated with spontaneous fermentation of Cabernet Sauvignon wines from Shangri-La wine region, China**

Yue Zhao^1^, Qingyang Sun^1^, Shusheng Zhu^1^, Fei Du^1^, Ruzhi Mao^2^, Lijing Liu^2,3^, Bin Tian^4*^ and Yifan Zhu^1,2,3*^

^1^ College of Plant Protection, Yunnan Agricultural University, Kunming, 650201, China

^2^ College of Food Science and Technology, Yunnan Agricultural University, Kunming, 650201, China

^3^ University Engineering Research Center for Grape & Wine of Yunan Province, Yunnan Agricultural University, Kunming, 650201, China

^4^ Faculty of Agriculture and Life Sciences, Lincoln University, Lincoln, 7647, New Zealand

*corresponding [yifanzhuyy@163.com](mailto:yifanzhuyy@163.com); [bin.tian@lincoln.ac.nz](mailto:bin.tian@lincoln.ac.nz)

**Supplementary Table**

| Isolate  No. | Fragment size of the representative yeasts and their Gen Bank accession numbers | | | | D1/D2 domain of 26S rRNA sequences | | ITS1-5.8S-ITS2 rRNA sequence | |
| --- | --- | --- | --- | --- | --- | --- | --- | --- |
|  | Size (bp)  (D1/D2 domain) | Accession No.  (D1/D2 domain) | Size (bp)  (ITS region) | Accession No.  (ITS region) | Type strain | Sequences accession number of type strain  (E-value)/identity% | Type strain | Sequences accession number of type strain  (E-value)/identity% |
| SN-5 | 585 | MN371953 | 555 | MN371884 | *Aureobasidium pullulans*  CBS 100524 T | FJ150952.1  (0.0)/99.64% | *Aureobasidium pullulans*  CBS 100524 T | FJ150905.1  (0.0)/98.55% |
| BZL-122 | 585 | MN371933 | 440 | MN371904 | *Brettanomyces bruxellensis*  CBS 72 T | AY964182.1  (0.0)/99.47% | *Brettanomyces bruxellensis*  CBS 72 T | KY103308.1  (0.0)/97.44% |
| DR-80 | 597 | MN371939 | 856 | MN371898 | *Candida glabrata*  CBS 138 T | AY497670.1  (0.0)/99.50% | *Candida glabrata*  CBS 138 T | CBS 138_ex19094_24524_ITS  (0.0)/99.12% |
| AD-1 | 579 | MN371919 | 599 | MN371918 | *Candida oleophila*  CBS 2219 T | NG_060820.1  (0.0)/99.65% | *Candida oleophila*  CBS 2219 T | AY528671.1  (0.0)/99.81% |
| XD-1-2 | 574 | MN371957 | 599 | MN371881 |  | NG_060820.1  (0.0)/99.30% |  | AY528671.1  (0.0)/99.24% |
| AD-52 | 583 | MN371924 | 605 | MN371913 | *Debaryomyces hansenii*  CBS 1098 | NG_055701.1  (0.0)/99.62% | *Debaryomyces hansenii*  CBS 1098 T | EU816279.1  (0.0)/99.26% |
| SN-41 | 582 | MN371956 | - | - |  | NG_055701.1  (0.0)/99.43% |  | - |
| BZL-89 | 586 | MN371931 | 723 | MN371906 | *Hanseniaspora opuntiae*  CBS 8733 T | NG_055312.1  (0.0)/99.12% | *Hanseniaspora opuntiae*  CBS 8733 T | AJ512435.1  (0.0)/99.23% |
| NT-52 | 577 | MN371949 | 718 | MN371888 | *Hanseniaspora uvarum*  CBS 104 T | KY107838.1  (0.0)/98.96% | *Hanseniaspora uvarum*  CBS 104 T | KY103563.1  (0.0)/99.57% |
| XD-23 | 586 | MN371960 | 685 | MN371878 |  | KY107838.1  (0.0)/99.15% |  | KY103563.1  (0.0)/85.58% |
| LTJ-24 | 587 | MN371944 | 718 | MN371893 |  | KY107838.1  (0.0)/99.13% |  | KY103563.1  (0.0)/99.14% |
| SN-17 | 587 | MN371954 | 708 | MN371883 |  | KY107838.1  (0.0)/99.65% |  | KY103563.1  (0.0)/99.28% |
| AD-49 | 580 | MN371923 | 723 | MN371914 |  | KY107838.1  (0.0)/99.66% |  | KY103563.1  (0.0)/99.15% |
| BZL-88 | 578 | MN371930 | 721 | MN371907 |  | KY107838.1  (0.0)/99.65% |  | KY103563.1  (0.0)/99.71% |
| DR-40 | 586 | MN371938 | 721 | MN371899 |  | KY107838.1  (0.0)/98.98% |  | KY103563.1  (0.0)/94.60% |
| XD-102 | 584 | MN371962 | 713 | MN371876 | *Hanseniaspora vineae*  NRRL Y-17529 T | NG_055415.1  (0.0)/98.76% | *Hanseniaspora vineae*  NRRL Y-17529 T | NR_138203.1  (0.0)/99.22% |
| BZL-111 | 583 | MN371932 | 713 | MN371905 |  | NG_055415.1  (0.0)/98.58% |  | NR_138203.1  (0.0)/98.13% |
| AD-7 | 518 | MN371920 | 347 | MN371917 | *Metschnikowia fructicola*  GXZJD32 | KC160603.1  (0.0)/98.18% | *Metschnikowia fructicola*  AP47 | FJ919773.1  (2e-128)/96.32% |
| SN-25 | 511 | MN371955 | 347 | MN371882 | *Metschnikowia pulcherrima*  CBS 610 T | CBS 610_ex32092_26279_LSU  (0.0)/98.41% | *Metschnikowia pulcherrima*  CBS 610 T | cr-CBS 610-ITS rRNA sequence from CBS culture collection  (2e-110)/92.28% |
| AD-58 | 586 | MN371925 | 578 | MN371912 | *Meyerozyma guilliermondii*  BCS 2030T | AY497675.1  (0.0)/100.00% | *Meyerozyma guilliermondii*  BCS 2030 T | NR_111247.1  (0.0/99.45% |
| LTJ-8 | 607 | MN371943 | 505 | MN371894 | *Papiliotrema flavescens*  (*Cryptococcus flavescens*)  CBS 942 T | AB035042.1  (0.0)/99.12% | *Papiliotrema flavescens*  (*Cryptococcus flavescens*)  CBS 942 T | AB035046.1  (0.0)/99.09% |
| XD-10-1 | 583 | MN371958 | 414 | MN371880 | *Pichia kluyveri*  NRRL Y-11519 T | U75727.1  (0.0)/98.19% | *Pichia kluyveri*  CBS 188 T | NR_138210.1  (0.0/99.23% |
| AD-17 | 572 | MN371922 | 417 | MN371915 |  | U75727.1  (0.0)/99.82% |  | NR_138210.1  4e-153/91.36% |
| NT-108 | 569 | MN371951 | 481 | MN371886 | *Pichia kudriavzevii*  CBS 5147 T | CBS 5147 ex 32128_42108_LSU  (0.0)/99.82% | *Pichia kudriavzevii*  CBS 5147 T | KY104577.1  (0.0)/99.58% |
| DR-125 | 578 | MN371941 | 482 | MN371896 |  | CBS 5147 ex 32128_42108_LSU  (0.0)/99.65% |  | KY104577.1  (0.0)/95.12% |
| NT-3 | 571 | MN371946 | 419 | MN371891 | *Pichia occidentalis*  (*Issatchenkia occidentalis*)  CBS 1910 T | KY108907.1  (0.0)/99.12% | *Pichia occidentalis*  (*Issatchenkia occidentalis*)  CBS 1910 T | cr-CBS 1910-ITS rRNA sequence from CBS culture collection  (0.0)/98.98% |
| XD-10-2 | 562 | MN371959 | 418 | MN371879 |  | KY108907.1  (0.0)/99.82% |  | cr-CBS 1910-ITS rRNA sequence from CBS culture collection  (0.0)/98.98% |
| BZL-82 | 571 | MN371929 | 421 | MN371908 |  | KY108907.1  (0.0)/99.64% |  | cr-CBS 1910-ITS rRNA sequence from CBS culture collection  (0.0)/98.98% |
| DR-11 | 572 | MN371937 | 424 | MN371900 |  | KY108907.1  (0.0)/99.65% |  | cr-CBS 1910-ITS rRNA sequence from CBS culture collection  (0.0)/98.26% |
| NT-22 | 569 | MN371947 | 391 | MN371890 | *Pichia terricola*  (*Issatchenkia terricola*)  BCS 2617 T | KY108920.1  (0.0)/99.45% | *Pichia terricola*  (*Issatchenkia terricola*)  BCS 2617 T | KY104650.1  (0.0)/99.22% |
| AD-9 | 582 | MN371921 | 572 | MN371916 | *Rhodotorula glutinis*  CBS 20 T | KX771201.1  (0.0)/99.46% | *Rhodotorula glutinis*  CBS 20 T | AF444539.1  (0.0)/99.29% |
| LTJ-6 | 591 | MN371942 | 572 | MN371895 |  | KX771201.1  (0.0)/99.64% |  | AF444539.1  (0.0)/98.95% |
| BZL-28 | 580 | MN371928 | 575 | MN371909 | *Saccharomycopsis vini*  CBS 4110 T | KY109519.1  (0.0)/99.65% | *Saccharomycopsis vini*  CBS 4110 T | KY105291.1  (0.0)/99.14% |
| BZL-14 | 498 | MN371927 | 430 | MN371910 | *Starmerella apicola*  (*Candida apicola*)  CBS 2868 T | CBS 2868 ex no 10264  (0.0)/98.16% | *Starmerella apicola*  (*Candida apicola*)  NRRL Y-2481 T | EU926482.1  (0.0)/99.25% |
| BZL-11 | 546 | MN371926 | 377 | MN371911 | *Saturnispora diversa*  CBS 4074 T | KY109545.1  (0.0)/99.63% | *Saturnispora diversa*  CBS 4074 T | KY105316.1  (0.0)/98.67% |
| NT-51 | 495 | MN371948 | 431 | MN371889 | *Starmerella bacillaris*  (*Candida zemplinina*)  CBS 9494 T | KY106894.1  (0.0)/99.59% | *Starmerella bacillaris*  (*Candida zemplinina*)  CBS 9494 T | KY102524.1  (0.0)/98.18% |
| XD-62 | 491 | MN371961 | 429 | MN371877 |  | KY106894.1  (0.0)/99.38% |  | KY102524.1  (0.0)/99.45% |
| LTJ-69 | 493 | MN371945 | 433 | MN371892 |  | KY106894.1  (0.0)/99.79% |  | KY102524.1  (0.0)/98.70% |
| BZL-128 | 494 | MN371934 | 429 | MN371903 |  | KY106894.1  (0.0)/99.59% |  | KY102524.1  (0.0)/99.18% |
| DR-8 | 498 | MN371936 | 432 | MN371901 |  | KY106894.1  (0.0)/99.18% |  | KY102524.1  (0.0)/98.93% |
| NT-69 | 491 | MN371950 | 441 | MN371887 | *Starmerella stellata*  (*Candida stellata*)  CBS 157 T | NG060828.1  (0.0)/98.37% | *Starmerella stellata*  (*Candida stellata*)  CBS 157 T | AY160766.1  (0.0)/95.60% |
| BZL-163 | 586 | MN371935 | 773 | MN371902 | *Torulaspora delbrueckii*  CBS 1146 T | NG_058413.1  (0.0)/99.15% | *Torulaspora delbrueckii*  CBS 1146 T | KY105617.1  (0.0)/99.34% |
| DR-110 | 577 | MN371940 | 591 | MN371897 | *Wickerhamomyces anomalus*  CBS 133 T | AF330114.1  (0.0)/99.82% | *Wickerhamomyces anomalus*  CBS 133 T | KY105876.1  (0.0)/97.83% |
| NT-110 | 588 | MN371952 | 713 | MN371885 | *Zygosaccharomyces bailii*  CBS 680 T | KY110234.1  (0.0)/99.48% | *Zygosaccharomyces bailii*  CBS 680 T | KY106020.1  (0.0)/91.99% |

**Table S1.** A list of non-*Saccharomyces* yeast isolates obtained from Shangri-La wine region with their identification results, fragment size, and GenBank accession number of D1/D2 regions and ITS regions.

*T*: type strains, *CBS*: Centraalbureau voor Schimmelcultures, *NRRL*: Agricultural Research Service Culture Collection

Part sequences of D1/D2 and ITS region of type strain were obtained from the database of CBS website.

Due to improper storage, isolate SN-41 was lost its activity before the experiment of ITS region amplification
